# Associations of food choice values and food literacy with overall diet quality: a nationwide cross-sectional study in Japanese adults

**DOI:** 10.1017/S000711452300082X

**Published:** 2023-11-28

**Authors:** Kentaro Murakami, Nana Shinozaki, M. Barbara E. Livingstone, Xiaoyi Yuan, Ryoko Tajima, Mai Matsumoto, Shizuko Masayasu, Satoshi Sasaki

**Affiliations:** 1 Department of Social and Preventive Epidemiology, School of Public Health, University of Tokyo, Tokyo 113-0033, Japan; 2 Nutrition Innovation Centre for Food and Health (NICHE), School of Biomedical Sciences, Ulster University, Coleraine BT52 1SA, UK; 3 Department of Nutritional Epidemiology and Shokuiku, National Institute of Biomedical Innovation, Health and Nutrition, Tokyo 162-8636, Japan; 4 Ikurien-naka, Ibaraki 311-0105, Japan

**Keywords:** Food literacy, Values, Knowledge, Skill, Diet quality, Sex, Gender

## Abstract

To date, a limited number of studies have examined aspects of food choice values and food literacy in relation to some aspects of dietary behaviours. The aim of this cross-sectional study was to comprehensively examine the associations of food choice values and food literacy with diet quality. In total, 2231 Japanese adults aged 19–80 years completed questionnaires asking about food choice values (accessibility, convenience, health/weight control, tradition, sensory appeal, organic, comfort and safety) and food literacy characterised by nutrition knowledge, cooking skills, food skills and eating behaviours (hunger, food responsiveness, emotional overeating, enjoyment of food, satiety responsiveness, emotional undereating, food fussiness and slowness in eating). As a measure of diet quality, the Healthy Eating Index-2015 (HEI-2015) was calculated using a brief-type diet history questionnaire (BDHQ) or a food combination questionnaire (FCQ). In males, after adjustment for potential confounding factors (including age, BMI and the ratio of reported energy intake to estimated energy requirement), the HEI-2015 derived from BDHQ and that derived from FCQ were associated significantly (*P* ≤ 0·02) and positively with the food choice values of organic and inversely with food fussiness. In females, the HEI-2015 showed positive associations with the food choice values of health/weight control, nutrition knowledge and cooking skills and an inverse association with food fussiness, irrespective of the dietary assessment questionnaire (*P* ≤ 0·03). In conclusion, this study suggests that several aspects of food choice values and food literacy were associated with diet quality, and the aspects related differed between males and females.

According to estimates in the Global Burden of Disease Study, suboptimal dietary intakes account for 22 % of total deaths and 15 % of disability-adjusted life years annually^(1)^ and exceed those of any other risk factor including tobacco smoking^(2)^. Because of the complex and varied nature of individual characteristics that are related to dietary behaviours^(3)^, an understanding of the factors that shape food choices and eating behaviours and thus determine the quality of diet is imperative^(4)^. According to the food choice process model^(5)^, food choice values are supposed to represent the proximal influences on food choice and eating behaviours, conveying the effects of more distal determinants, including life course factors (such as socio-economic factors), socio-cultural resources and cognitive resources^(4)^.

Another relevant concept that has emerged recently is that of food literacy. Although there are several definitions of food literacy^(6–8)^, the most widely cited definition is that developed by Vidgen and Gallegos^(9)^, in which food literacy is described as ‘a collection of inter-related knowledge, skills and behaviours required to plan, manage, select, prepare and eat food to meet needs and determine intake’^(9)^. Thus, food literacy is not just concerned with nutrition knowledge but also includes skills and behaviours, from knowing where food comes from to the ability to select and prepare these foods and behave in ways that meet dietary guidelines^(10)^.

To date, only a limited number of studies have examined aspects of food choice values and food literacy in relation to some aspects of dietary behaviours^(11–20)^. For example, a survey of Finnish adults suggests that the less healthy dietary habits (such as low consumption of vegetables/fruit and high consumption of energy-dense foods) are partly attributable to the higher priority of motives related to price and familiarity and the lower priority of health motives^(11)^. Several studies have also shown that a composite measure of food literacy is associated with a higher consumption of vegetables, fruit and fish and a lower consumption of sugar-sweetened beverages in Dutch adults^(12)^ and a higher score of simple diet quality score in Korean adults^(13)^. Further, a relatively consistent positive association between nutrition knowledge and diet quality was observed^(14,15,18,19)^, with some exceptions^(16)^. Conversely, findings on cooking skills and food skills seem inconsistent. In an Australian sample consisting of predominantly females (80 %) with relatively high education and socio-economic status, a simple measure of diet quality was positively associated with food skills, but not with cooking skills^(14)^, whereas in a nationally representative sample of Irish adults, cooking skills, but not food skills, appeared to be associated with some aspects of healthy dietary behaviours such as lower saturated fat intake^(15)^. For eating behaviours, a prospective cohort study among children showed that a higher score on food fussiness (i.e. tendency to refuse new foods at first) and a lower score on the enjoyment of food were associated with a lower quality of the diet^(20)^. To our knowledge, however, no research has comprehensively examined food choice values and food literacy in relation to overall diet quality in free-living settings.

Additionally, some of the previous studies mentioned have not considered even fundamental factors such as sex and age^(19)^ or have relied on relatively crude or unvalidated measures of diet quality^(12,13,15)^. Furthermore, almost all previous studies have been conducted in Western countries^(11,12,14–20)^. Conversely, research in Asian countries, including Japan, where dietary habits are considerably different from those in Western countries^(21–27)^, is limited^(13)^. Therefore, the aim of the present cross-sectional study was to comprehensively examine food choice values and food literacy (characterised by nutrition knowledge, cooking and food skills, and eating behaviours) in relation to the nutritional quality of the overall diet in a nationwide sample of free-living Japanese adults. On the basis of previous studies^(11–20)^, we hypothesised that several aspects of food choice values (such as health and accessibility) and food literacy (such as nutrition knowledge, cooking skills, food skills, food fussiness and enjoyment of food) are associated with diet quality. We also hypothesised that the association would be clearer for females than males, since it is usually females who are in charge of food preparation in Japanese households^(28)^.

## Methods

### Study procedure and participants

Detailed descriptions of the study procedure and participants are available elsewhere^(29)^. Briefly, between October and December 2018, a questionnaire survey was conducted in thirty-two prefectures, where the residents account for > 85 % of the total population of Japan. The target population consisted of adult participants in the MINNADE (MINistry of health, labour and welfare-sponsored Nationwide study on Dietary intake Evaluation) study, a dietary record survey^(30)^. This was because the present study was originally designed as an add-on to the MINNADE study; however, the use of data obtained within the MINNADE study has not yet been permitted by the Ministry of Health, Labour and Welfare, Japan^(30)^. Potential participants were apparently healthy Japanese adults living in private households in Japan. Exclusion criteria were dietitians, individuals living with a dietitian, those working with a research dietitian, those who had received dietary counselling from a doctor or dietitian, those taking insulin treatment for diabetes, those receiving dialysis treatment and pregnant or lactating women. Participation of only one person per household was permitted. These criteria were made in accordance with those in the MINNADE study, the ultimate purpose of which was to describe nationwide data on dietary characteristics and eating behaviours in Japan^(30)^.

Recruitment of participants and data collection were conducted by our research dietitians (*n* 476). The non-random sampling procedure was performed to reflect the proportion of the overall Japanese population in each region but with the intention to recruit an equal number of males and females. Of 2983 adult participants in the MINNADE study, 2248 individuals participated in the present study (response rate: 75 %). For analysis, we excluded participants with missing information related to the variables of interest (*n* 5) and those aged outside the 19–80 years age range (*n* 12), leaving 2231 participants aged 19–80 years. All information was collected by questionnaires specially designed for this survey. Responses to all questions (except for those regarding nutrition knowledge) were checked by staff at the study centre. If any responses were missing, the participant was asked to complete the questions again in person or by telephone.

The study was conducted according to the guidelines laid out in the Declaration of Helsinki, and all procedures involving human subjects were approved by the Ethics Committee of the University of Tokyo Faculty of Medicine (protocol code: 12 031; date of approval: 17 July 2018). Written informed consent was obtained from each participant and from a parent or guardian for participants aged < 20 years.

### Assessment of food choice values

Food choice values were assessed using the Japanese version of the food choice values scale^(4)^. As detailed descriptions on the development process of the Japanese version (as well as for other instruments shown below) are available elsewhere^(29)^, only a brief description is provided here. In short, the food choice values scale is a twenty-five-item, self-administered questionnaire measuring eight factors of food choice values: accessibility, convenience, health/weight control, tradition, sensory appeal, organic, comfort and safety^(4)^. Participants were asked to answer how important each item is when deciding what foods to buy or eat on a daily basis. The possible responses, based on a Likert scale, ranged from 1 to 5 (1: not at all, 2: a little, 3: moderately, 4: quite a bit and 5: very). The score for each factor was calculated by the sum of the scores divided by the number of items (four items for organic and three items for others), with possible scores ranging from 1 to 5. In the present sample, Cronbach’s *α* for the assessment of internal consistency ranged from 0·61 (sensory appeal) to 0·87 (convenience)^(29)^, which was comparable to observations in previous studies (range: 0·54–0·89)^(4,31)^.

### Assessment of food literacy

In this study, food literacy was characterised by nutrition knowledge, cooking and food skills, and eating behaviours, in accordance with the most widely used description of food literacy: ‘a collection of inter-related knowledge, skills and behaviours required to plan, manage, select, prepare and eat food to meet needs and determine intake’^(9)^.

#### Nutrition knowledge

Nutrition knowledge was assessed using the Japanese general nutrition knowledge questionnaire (JGNKQ); the structure, validity and reliability of the JGNKQ have been described elsewhere^(32)^. Briefly, the original version of the JGNKQ is a 147-item, self-administered questionnaire consisting of five sections: dietary recommendations, sources of nutrients, choosing everyday foods, diet–disease relationships and reading a food label. The JGNKQ used in this study was a 143-item version in which four items with a very low prevalence of correct answers in the original version were removed. For each item, the correct response was assigned 1 point, whereas an incorrect or missing response was assigned 0 point. Thus, the possible total score ranged from 0 to 143, with a higher score reflecting a higher level of nutrition knowledge. In the present sample, Cronbach’s *α* for the 143 items was 0·96^(29)^, which was comparable to that observed in the development process of the JGNKQ (0·95)^(32)^.

#### Cooking and food skills

Cooking skills and food skills were assessed using the Japanese version of the English scale for cooking and food skills, a self-administered questionnaire^(33)^. Briefly, questions on cooking skills (*n* 14) ask about cooking methods and food preparation techniques, whereas questions on food skills (*n* 19) ask about meal planning and preparation, shopping, budgeting, resourcefulness and label reading/consumer awareness. Participants were asked to rate how well they felt they performed each of the skills described according to a seven-point Likert scale (1: very poor, 7: very good). An option of ‘never/rarely do it’ was also available for participants who considered that a skill is not used; a score of 0 was assigned when this response was selected. The scores of cooking skills and food skills were calculated as the sum of all the items; thus, possible scores ranged from 0 to 98 for cooking skills and from 0 to 133 for food skills. In the present sample, Cronbach’s *α* was 0·95 for the fourteen cooking skill items and 0·96 for the nineteen food skill items^(29)^, which was higher than those observed in the original study (range: 0·78–0·94)^(33)^.

#### Eating behaviours

Eating behaviours were assessed using the Japanese version of the Adult Eating Behavior Questionnaire (AEBQ) prepared based on the original English version^(34)^. Briefly, the AEBQ is a thirty-five-item, self-administered questionnaire, measuring four food approach scales, namely hunger (five items), food responsiveness (four items), emotional overeating (five items) and enjoyment of food (three items), as well as four food avoidance scales, namely satiety responsiveness (four items), emotional undereating (five items), food fussiness (five items) and slowness in eating (four items)^(34)^. Item responses were rated based on a five-point Likert scale ranging from ‘strongly disagree’ to ‘strongly agree’, and a mean score was calculated for each scale (possible score ranging from 1 to 5). In the present sample, Cronbach’s *α* ranged from 0·65 (slowness in eating) to 0·89 (emotional undereating)^(29)^, which was comparable to those observed in previous studies (range: 0·67–0·97)^(34–38)^.

### Dietary assessment

Information on dietary habits during the preceding month was assessed using a brief-type diet history questionnaire (BDHQ), details of which have been described elsewhere^(39,40)^. In brief, the BDHQ is a four-page self-administered questionnaire which consists of structured questions asking about the consumption frequency of selected foods commonly consumed in Japan, as well as general dietary behaviour and usual cooking methods. Estimates of daily intake of foods (fifty-eight items in total), energy and selected nutrients were calculated using an *ad hoc* computer algorithm for the BDHQ. This algorithm incorporates the sex-specific portion size, determined mainly based on recipe books for Japanese dishes^(39)^, and nutrient composition of each food item derived from the 2015 version of the Standard Tables of Food Composition in Japan^(41)^.

Information on dietary habits during the preceding month was also assessed using a food combination questionnaire (FCQ), a four-page self-administered questionnaire. Details of the FCQ’s development process, structure, content and algorithms for dietary intake calculation have been published elsewhere^(26)^. Briefly, in the FCQ, questions on consumption frequency of staple foods (as the number of days per week) are followed by questions on relative consumption frequency of accompanying foods (‘always’, ‘sometimes’ and ‘never’) for each meal type (i.e. breakfast, lunch, dinner and snacks). These were determined based on the most commonly consumed combinations of seventeen selected food groups identified in the 16-d weighed dietary record data collected from 242 Japanese adults aged 31–81 years^(27)^ using the frequent item sets data mining methods^(42)^. On the basis of a series of *ad hoc* computer algorithms in the FCQ^(26,27)^ and the 2015 version of the Standard Tables of Food Composition in Japan^(41)^, estimated intakes of food groups, energy and selected nutrients were calculated. The calculation was done for each meal type, and the overall intake was calculated as the sum of the intake of each meal type.

### Calculation of diet quality score

In the present study, we used the Healthy Eating Index 2015 (HEI-2015)^(43–45)^ as a measure of diet quality. The HEI-2015 is a 100-point scale to assess compliance with the 2015–2020 Dietary Guidelines for Americans^(46)^, with a higher score indicating a better quality of overall diet. The HEI-2015 consists of nine adequacy components (total fruits, whole fruits, total vegetables, greens and beans, whole grains, dairy products, total protein foods, seafood and plant proteins, and fatty acids as the ratio of the sum of PUFA and MUFA to SFA) and four moderation components (refined grains, Na, added sugars and saturated fats). The efficacy of the HEI-2015 in assessing the overall diet quality of Japanese has been supported by our previous analyses: a higher total score in the HEI-2015 was associated with favourable patterns of the overall diet, including higher intakes of dietary fibre and key vitamins and minerals and lower intakes of saturated fats, added sugars and Na^(47,48)^.

For both dietary assessment tools (i.e. BDHQ and FCQ), component scores needed for the calculation of HEI-2015 were calculated using the Japanese version^(49)^ of the US Food Patterns Equivalents Database^(50)^, except for fatty acids and Na, for which the 2015 version of the Standard Tables of Food Composition in Japan^(41)^ was used. As described in detail elsewhere^(49)^, we calculated the HEI-2015 component scores based on energy-adjusted values of overall dietary intake, namely amount per 1000 kcal of energy or percentage of energy, except for fatty acids, and then we summed up these scores to obtain the HEI-2015 (total) score. We derived the HEI-2015 score for total diet from both the BDHQ and the FCQ.

The relative validity of the BDHQ and FCQ for estimating the HEI-2015 has been examined. Briefly, the Pearson correlation coefficient between the BDHQ and a 16-d weighed dietary record was 0·52 in women (*n* 121) and 0·43 in men (*n* 121)^(49)^. The Pearson correlation coefficient between the FCQ and a 4-d weighed dietary record was 0·50 among both 111 women and 111 men^(51)^.

### Assessment of basic characteristics

In this study, sex was self-reported. Age at the time of the study was calculated based on birth date of the participant and the date the questionnaires were answered. Self-reported information on body height and weight was obtained, based on which BMI was calculated (in kg/m^2^). We also calculated the ratio of reported energy intake (derived from the BDHQ or FCQ) to estimated energy requirement (as a measure of overall accuracy of dietary reporting). For each participant, estimated energy requirement was calculated based on sex-specific equations published from the US Dietary Reference Intakes^(52)^, using information on age, body height, body weight and physical activity. We assumed ‘low active’ level of physical activity (i.e. physical activity level ≥ 1·4 to < 1·6) for all participants during this calculation not only because of a lack of information on physical activity in the present study but also because another nationwide study in 7000 adults aged 20–69 years showed a large proportion of the participants (69 %) categorised into ‘low active’ level of physical activity^(53)^.

### Statistical analysis

All analyses were conducted for males and females separately; this a priori decision was based on large sex differences in mean values of food choice values and food literacy in this population^(29)^. Descriptive data are presented as means and standard deviations. As the main analysis, we examined the associations of food choice values and food literacy (characterised by nutrition knowledge, cooking and food skills, and eating behaviours) with diet quality (as assessed using the HEI-2015 derived from the BDHQ or FCQ) using sex-specific multiple linear regression models. All the variables of interest were included in a model simultaneously. We also included age, BMI and the ratio of energy intake (derived from the BDHQ or FCQ) to estimated energy requirement as covariates on the basis of the following reasons. First, these variables were associated with the explanatory variables in this population; see Murakami *et al.* (2022)^(29)^ for age and BMI and Pearson’s correlation coefficients for the ratio of energy intake to estimated energy requirement ranging from −0·24 (FCQ’s ratio and enjoyment of food for females) to 0·28 (FCQ’s ratio and slowness in eating for males). Second, our previous Japanese studies have shown that these variables were also associated with diet quality^(47,48,54)^. The variance inflation factor scores for any variable in any model (range: 1·11–3·91) were within acceptable limits (< 10)^(55)^, suggesting that multicollinearity was not an issue. Regression coefficients (*β*) were calculated as the change of the HEI-2015 with 1-sd increase of each variable. In this study, the estimates of HEI-2015 from the BDHQ and FCQ did not correlate well (Pearson’s correlation coefficient: 0·50 for males and 0·48 for females). Furthermore, as noted above, the correlation between the HEI-2015 by the BDHQ and that by the 16-d weighed dietary record was mediocre at best^(49)^, as was the correlation between the HEI-2015 by the FCQ and that by the 4-d weighed dietary record^(51)^; thus, external validation did not provide any reassurance. Since choosing which is the better indicator of diet quality in this study is difficult, we focused only on the consistently observed associations regardless of the diet assessment questionnaire. All statistical analyses were performed using SAS statistical software (version 9.4, SAS Institute Inc.). We considered two-tailed *P* values < 0·05 statistically significant.

## Results

The present analysis included 1068 males and 1163 females aged 19–80 years, with a mean age of 50 years (Table 1). The mean BMI (kg/m^2^) was 23·7 (sd 3·3) for males and 22·3 (sd 3·5) for females. The mean values of HEI-2015 among males were 55·5 (sd 6·5) based on the BDHQ and 53·0 (sd 2·8) based on the FCQ. The corresponding values were 57·2 (sd 6·6) and 53·6 (sd 2·6) among females.

**Table 1.** Basic characteristics of the study population (*n* 2231)

	Male (*n* 1068)		Female (*n* 1163)	
	Mean	sd	Mean	sd
Age (years)	50·4	17·2	50·0	17·5
Body height (cm)*	169·4	6·3	156·3	5·9
Body weight (kg)*	68·0	10·9	54·4	9·0
BMI (kg/m^2^)†	23·7	3·3	22·3	3·5
EER (kJ/d)	10 087	1142	7846	828
EI (kJ/d)				
BDHQ	8517	2305	6941	1839
FCQ	6857	910	6986	781
EI:EER				
BDHQ	0·86	0·26	0·90	0·27
FCQ	0·69	0·13	0·90	0·15
Healthy Eating Index-2015‡				
BDHQ	55·5	6·5	57·2	6·6
FCQ	53·0	2·8	53·6	2·6

BDHQ, brief-type diet history questionnaire; EI, energy intake; EER, estimated energy requirement; FCQ, food combination questionnaire.

* Based on self-report.

† Calculated using self-reported body height and weight.

‡ A maximum score is 100. A higher score indicates a higher diet quality.

Table 2 shows associations of the HEI-2015 (derived from the BDHQ and FCQ) with food choice values and food literacy characterised by nutrition knowledge, cooking and food skills, and eating behaviours in males. After adjustment for potential confounding factors (including age, BMI and the ratio of reported energy intake to estimated energy requirement), the HEI-2015 derived from the BDHQ was significantly positively associated with the food choice values of health/weight control and organic, nutrition knowledge, and slowness in eating and inversely with food fussiness. The change of the HEI-2015 per 1 sd increase of each variable was +0·82 (se 0·23) for the food choice values of health/weight control, +0·82 (se 0·34) for the food choice values of organic, +0·49 (se 0·19) for nutrition knowledge, +0·52 (se 0·19) for slowness in eating and –1·17 (se 0·20) for food fussiness. When the HEI-2015 derived from the FCQ was examined, there were positive associations with the food choice values of organic and food skills and inverse associations with the food choice values of accessibility, cooking skills and food fussiness. The change of the HEI-2015 per 1 sd increase of each variable was +0·51 (se 0·15) for the food choice values of organic, +0·35 (se 0·15) for food skills, –0·26 (se 0·11) for the food choice values of accessibility, –0·38 (se 0·14) for cooking skills and –0·42 (se 0·09) for food fussiness.

**Table 2.** Associations of the HEI-2015 assessed by the BDHQ and FCQ with food choice values and food literacy characterised by nutrition knowledge, cooking and food skills, and eating behaviours in 1068 males

			HEI-2015 based on BDHQ*			HEI-2015 based on FCQ*		
	Mean	sd	*β* †	se †	*P*	*β* †	se †	*P*
Food choice values‡								
Accessibility	3·06	0·84	–0·27	0·24	0·25	–0·26	0·11	0·01
Convenience	2·94	0·91	–0·26	0·23	0·26	0·08	0·10	0·42
Health/weight control	2·67	0·95	0·82	0·23	0·0005	0·13	0·10	0·23
Tradition	1·98	0·75	–0·14	0·22	0·53	0·09	0·10	0·34
Sensory appeal	3·17	0·73	0·20	0·22	0·37	0·08	0·10	0·44
Organic	2·72	0·87	0·82	0·34	0·02	0·51	0·15	0·0007
Comfort	2·19	0·82	0·06	0·23	0·78	–0·12	0·10	0·23
Safety	3·14	0·95	–0·03	0·31	0·91	–0·14	0·14	0·32
Nutrition knowledge§	63·9	25·8	0·49	0·19	0·009	0·10	0·08	0·25
Cooking and food skills								
Cooking skills||	30·3	25·9	–0·24	0·32	0·44	–0·38	0·14	0·007
Food skills¶	43·6	34·1	–0·05	0·33	0·89	0·35	0·15	0·02
Eating behaviours‡								
Hunger	2·67	0·66	–0·14	0·21	0·50	0·09	0·09	0·32
Food responsiveness	2·58	0·64	–0·27	0·23	0·24	–0·13	0·10	0·21
Emotional overeating	2·24	0·77	–0·05	0·20	0·82	–0·06	0·09	0·54
Enjoyment of food	3·94	0·76	0·08	0·21	0·71	0·00	0·09	0·97
Satiety responsiveness	2·46	0·66	–0·07	0·19	0·70	–0·04	0·09	0·61
Emotional undereating	2·56	0·90	0·10	0·19	0·59	–0·04	0·08	0·66
Food fussiness	2·63	0·78	–1·17	0·20	< 0·0001	–0·42	0·09	< 0·0001
Slowness in eating	2·44	0·72	0·52	0·19	0·007	0·05	0·09	0·54

*β*, regression coefficient; BDHQ, brief-type diet history questionnaire; FCQ, food combination questionnaire; HEI-2015, Healthy Eating Index-2015.

* A maximum score is 100. A higher score indicates a higher diet quality.

† Models with listed variables, age (years, continuous), BMI (kg/m^2^, continuous) and the ratio of energy intake (derived from the BDHQ or FCQ) to estimated energy requirement (continuous) as the explanatory variables and the HEI-2015 as the response variable; regression coefficients mean the change of HEI-2015 with 1-sd increase of each variable.

‡ Possible score ranging from 1 to 5 for each variable.

§ Possible score ranging from 0 to 143.

|| Possible score ranging from 0 to 98.

¶ Possible score ranging from 0 to 133.

In females (Table 3), the HEI-2015 derived from the BDHQ was positively associated with food choice values of health/weight control and safety, nutrition knowledge, and cooking skills and inversely with the food choice values of accessibility, emotional overeating and food fussiness. The change of the HEI-2015 per 1 sd increase of each variable was +0·79 (se 0·21) for the food choice values of health/weight control, +0·54 (se 0·27) for the food choice values of safety, +0·44 (se 0·17) for nutrition knowledge, +0·67 (se 0·25) for cooking skills, –0·43 (se 0·20) for the food choice values of accessibility, –0·42 (se 0·20) for emotional overeating and –0·47 (se 0·19) for food fussiness. When the HEI-2015 derived from the FCQ was examined, there were positive associations with the food choice values of health/weight control and organic, nutrition knowledge, and cooking skills and inverse associations with the food choice values of convenience and food fussiness. The change of the HEI-2015 per 1 sd increase of each variable was +0·22 (se 0·09) for the food choice values of health/weight control, +0·39 (se 0·13) for the food choice values of organic, +0·17 (se 0·07) for nutrition knowledge, +0·27 (se 0·11) for cooking skills, –0·20 (se 0·09) for the food choice values of convenience and –0·21 (se 0·08) for food fussiness.

**Table 3.** Associations of the HEI-2015 assessed by the BDHQ and FCQ with food choice values and food literacy characterised by nutrition knowledge, cooking and food skills, and eating behaviours in 1163 females

			HEI-2015 based on BDHQ*			HEI-2015 based on FCQ*		
	Mean	sd	*β* †	se †	*P*	*β* †	se †	*P*
Food choice values‡								
Accessibility	3·32	0·71	–0·43	0·20	0·03	–0·13	0·09	0·13
Convenience	3·23	0·76	–0·33	0·20	0·10	–0·20	0·09	0·02
Health/weight control	2·97	0·84	0·79	0·21	0·0001	0·22	0·09	0·01
Tradition	2·19	0·74	–0·35	0·21	0·11	–0·12	0·09	0·20
Sensory appeal	3·38	0·65	0·03	0·20	0·91	0·07	0·09	0·42
Organic	3·16	0·76	0·50	0·29	0·09	0·39	0·13	0·002
Comfort	2·45	0·79	0·01	0·21	0·97	–0·04	0·09	0·64
Safety	3·48	0·82	0·54	0·27	0·04	0·07	0·12	0·54
Nutrition knowledge§	76·0	21·8	0·44	0·17	0·01	0·17	0·07	0·03
Cooking and food skills								
Cooking skills||	55·2	19·5	0·67	0·25	0·006	0·27	0·11	0·01
Food skills¶	79·8	24·4	0·17	0·25	0·49	–0·05	0·11	0·67
Eating behaviours‡								
Hunger	2·87	0·72	–0·20	0·21	0·34	–0·06	0·09	0·49
Food responsiveness	2·88	0·67	0·07	0·23	0·74	–0·04	0·10	0·67
Emotional overeating	2·48	0·80	–0·42	0·20	0·03	–0·06	0·09	0·51
Enjoyment of food	4·09	0·72	–0·27	0·20	0·19	0·03	0·09	0·72
Satiety responsiveness	2·72	0·72	–0·19	0·18	0·31	–0·10	0·08	0·20
Emotional undereating	2·87	0·80	0·07	0·18	0·68	0·04	0·08	0·59
Food fussiness	2·54	0·77	–0·47	0·19	0·01	–0·21	0·08	0·009
Slowness in eating	2·69	0·70	0·22	0·17	0·20	0·09	0·07	0·21

*β*, regression coefficient; BDHQ, brief-type diet history questionnaire; FCQ, food combination questionnaire; HEI-2015, Healthy Eating Index-2015

* A maximum score is 100. A higher score indicates a higher diet quality.

† Models with listed variables, age (years, continuous), BMI (kg/m^2^, continuous) and the ratio of energy intake (derived from the BDHQ or FCQ) to estimated energy requirement (continuous) as the explanatory variables and the HEI-2015 as the response variable; regression coefficients mean the change of HEI-2015 with 1-sd increase of each variable.

‡ Possible score ranging from 1 to 5 for each variable.

§ Possible score ranging from 0 to 143.

|| Possible score ranging from 0 to 98.

¶ Possible score ranging from 0 to 133.

## Discussion

In this cross-sectional study, we found that in males, after adjustment for potential confounding factors, the HEI-2015 derived from BDHQ and that derived from FCQ were associated significantly and positively with the food choice values of organic and inversely with food fussiness. In females, the HEI-2015 showed positive associations with the food choice values of health/weight control, nutrition knowledge, and cooking skills and an inverse association with food fussiness, irrespective of the dietary assessment questionnaire. To our knowledge, this is the first study to comprehensively examine food choice values and food literacy (characterised by nutrition knowledge, cooking and food skills, and eating behaviours) in relation to overall diet quality.

Generally consistent with a previous study^(11)^, a positive association between the food choice values of health/weight control and diet quality was found for females in both BDHQ- and FCQ-based analyses but not for males (the positive association was only observed in the BDHQ-based analysis). Reasonable explanations for this finding may include the fact that females are primarily responsible for cooking and possibly grocery shopping in many Japanese households^(28)^, that females tend to be exposed to stronger sociocultural norms regarding body shape^(56)^, and that females are more involved and preoccupied with food than males^(57)^. Conversely, a positive association between the food choice values of organic and diet quality was found for males in both BDHQ- and FCQ-based analyses but not for females (the positive association was only observed in the FCQ-based analysis). A recent study showed that higher levels of organic food consumption were associated with healthier dietary patterns overall^(58)^, consistent with the present findings. It is unclear why the association was more evident in males than in females in the present study, but it may be that for males, choosing foods labelled ‘organic’ is an easy, recognisable and healthy option. Regarding the food choice values of accessibility (i.e. physical and financial ease of purchasing the product), we did not find consistent associations (inverse relations observed only in the FCQ- and BDHQ-based analyses in males and females, respectively). This might reflect the cautious dietary habits of the Japanese, such as low intake of sugar-sweetened beverages and infrequent snacking behaviours^(21,48,59,60)^.

We found a positive association between nutrition knowledge and diet quality in females, regardless of the dietary assessment questionnaire. This is consistent with the results from several previous studies^(14,15,18,19)^ but also is supported by a traditional hypothesis that an increase in nutrition knowledge improves attitudes towards healthy eating and subsequently improves eating behaviours (i.e. knowledge-attitude-behaviour model)^(61)^.

In relation to cooking skills, significant positive associations with diet quality were observed in females (for both BDHQ and FCQ). These findings are in line with a study which has shown that a high level of cooking skills is associated with a high consumption frequency of fruit and vegetables in Japanese females^(28)^. In addition, cooking identity (i.e. the degree to which someone sees himself or herself as a good cook), but not cooking skills, was independently and positively associated with a simple diet quality score in a study in Ireland^(15)^. An Australian study showed that cooking skills were positively correlated with a diet quality score and intakes of several healthy foods such as fruit and vegetables, although the association between cooking skills and the diet quality score did not reach statistical significance after adjustment for other factors such as sex, age and nutrition knowledge^(14)^. Conversely, we found no association between food skills and diet quality in any analyses in females, which may be because food skills are, on average, high in females in Japan^(29)^.

Among eating behaviours, we found that food fussiness was consistently and inversely associated with overall diet quality in both males and females. Often observed in children^(20)^, this appears plausible given that food fussiness may hinder access to healthy foods, lowering diet quality. On the other hand, no association between enjoyment of food and diet quality was found in any of the analyses, contrasting with previous findings in children^(20)^. The reason for this is unclear but may be due to the overall high enjoyment of food in this population^(29)^.

As hypothesised, the observed association was somewhat clearer for females than males. This may reflect that females are generally responsible for food preparation in Japanese households^(28)^, as mentioned above, as well as insufficient interest in nutrition, nutritional knowledge, cooking skills and food skills among males as a whole^(29)^. Furthermore, this may suggest that the correlates of diet quality are more difficult to understand among males than among females, considering a recent finding that Japanese males with lower cooking skills tended to be married and have a family member as the main meal preparer (wife and mother), while most Japanese females cooked by themselves irrespective of marital status^(28)^. In this regard, information about the living circumstance (e.g. responsibility for food selection and cooking) might be more fruitful.

The strengths of the present study include the simultaneous and comprehensive focus on food choice values and food literacy (nutrition knowledge, cooking and food skills, and eating behaviours) and the use of well-established scales for these variables (particularly nutrition knowledge) and diet quality (HEI-2015), as well as a large nationwide sample with almost the equal proportions for sex and age categories. However, there are also several limitations. First, the cross-sectional nature of this study does not permit the assessment of causality or its direction owing to the uncertain temporality of the association. Future research with prospective design is needed to confirm the present findings. Second, as described previously^(29)^, the present population, not a nationally representative sample of the Japanese, may have been biased towards greater health consciousness. Further research in a more representative sample is thus warranted. Third, the development process of the Japanese versions of assessment tools for food choice values, cooking and food skills, and eating behaviours did not consider cultural differences between Japan and Western countries. Consequently, these tools may not be optimal for use in the Japanese population, although the internal consistency of all the scores was comparable to that observed in previous studies. Fourth, although the assessment of diet quality was made using a well-established measure (HEI-2015) on the basis of validated tools (BDHQ and FCQ), measurement of dietary intake cannot be done without error. To minimise this issue, we focused only on the consistently observed associations regardless of the diet assessment questionnaire, as well as using a measure of the overall accuracy of dietary reporting (ratio of reported energy intake to estimated energy requirement) as a covariate. Finally, although we made adjustment for basic and important variables and all analyses were conducted for males and females separately, the possibility of residual confounding could not be ruled out. In particular, the present analysis could not consider any socio-economic variables because of a lack of information. However, while it is generally considered that education is a strong determinant of future employment and income and that knowledge and skills are attained through education^(62)^, nutrition knowledge was not significantly related to education or household income in a previous study of 1165 Japanese adults aged 18–64 years^(63)^. Further, previous Western studies have indicated that the associations of age and sex with food choice values^(11,64)^, except for values related to price cheapness of food^(57)^, as well as with cooking and food skills^(14,15)^, were stronger than those with education, although nutrition knowledge was strongly associated with education^(65–67)^. Taken together, it is unlikely that socio-economic factors entirely explain the findings observed here. Nevertheless, future research should incorporate the assessment of socio-economic variables to obtain more comprehensive pictures.

To conclude, in this nationwide cross-sectional study in Japan, several aspects of food choice values and food literacy were associated with diet quality, and the aspects related differed between males and females. Given that the selection, amount, combination of foods consumed and thus nutritional quality are markedly different between meal types (i.e. breakfast, lunch, dinner and snacks)^(21–27)^, a sensible next step would be to investigate if the associations of food choice values and food literacy with diet quality differ by meal types.
